# Upgrading the Nutritional Value of PKC Using a *Bacillus subtilis* Derived Monocomponent β-Mannanase

**DOI:** 10.3390/molecules27020563

**Published:** 2022-01-17

**Authors:** Luis-Miguel Gomez-Osorio, Janni Ulnits Nielsen, Helle Jakobe Martens, Reinhard Wimmer

**Affiliations:** 1 Alura Animal Health and Nutrition, Medellín 110921, Colombia; luis.gomez.osorio@alura.bio; 2 CIBAV Research Group, Facultad de Ciencias Agrarias, Universidad de Antioquia, Medellín 050034, Colombia; 3 Department of Chemistry and Bioscience, Aalborg University, Frederik Bajers vej 7H, 9220 Aalborg, Denmark; janniulnits@live.dk; 4 Section for Forest, Nature and Biomass, Department of Geosciences and Natural Resource Management, University of Copenhagen, Rolighedsvej 23, 1958 Frederiksberg, Denmark; hjm@ign.ku.dk

**Keywords:** palm kernel, β-mannanase, mannan, prebiotic, mannan oligosaccharides, cage effect, non-starch polysaccharides

## Abstract

Palm kernel cake (PKC) is an abundant side stream that can only be added to non-ruminant feed in small concentrations due to its content of antinutritional factors, mainly galactomannan, which cannot be digested by non-ruminants. β-mannanases can be added to partially hydrolyze galactomannan to form mannose oligosaccharides, which are known to be prebiotic. We here investigate the action of a β-mannanase from *B. subtilis* on PKC by colorimetry, NMR and fluorescence microscopy. The amount of mannan oligosaccharides in solution was significantly increased by the β-mannanase and their degree of polymerization (DP) was significantly reduced. There was a dose-response behavior in that larger β-mannanase concentrations increased the amount of soluble mannose oligosaccharides while reducing their average DP. Using confocal immunofluorescence microscopy, solubilization of galactomannan in PKC was clearly visualized. Images show a clear disruption of the cellulose and galactomannan structures of the PKC cell walls. We thus show in this study that using commercial dosages of β-mannanase on PKC can lead to formation of prebiotic compounds. Thus, this study suggests that utilization of PKC in poultry feed formulation might be increased by addition of a β-mannanase and would improve the return on investment.

## 1. Introduction

The past decades have seen a rising interest in supplementing animal feed formulations with byproducts from plant food production. This is in part due to a constant strain on the grain market in terms of high price volatility and availability and in part due to the growing concern for minimizing the carbon footprint of agriculture, where locally sourced byproducts are at an advantage over imported products [1]. One such byproduct is palm kernel cake (PKC) which remains after palm oil extraction by the expeller method. Countries such as West Africa, Indonesia, Malaysia and several African and Latin American countries produce large amounts of PKC from palm (*Elaeis guineensis*) nuts after oil removal [2]. With the export prices of palm oil from Asia soaring from an average of 660 USD/ton in February 2020 to 970 USD/ton, there is an increase in palm oil production and a concomitant increase in of the amount of resulting PKC and, thus, the potential gain of converting PKC to a high-value product increases [3]. Soybean meal (SBM) is used in animal feed and the production of SBM takes up valuable arable land. SBM must be purchased, imported and transported over large distances, while PKC is an inexpensive side-stream present in abundance. Therefore, replacing a part of SBM in animal feed by PKC would reduce the costs as well as the carbon footprint of animal husbandry, especially in regions with large amounts of PKC available. However, at present, PKC can only be used in small amounts as a substitute for soybean meal (SBM) in poultry nutrition due to the high fiber content which acts as an antinutritional factor (ANF). Therefore, processing of PKC to destroy the ANFs using physical, chemical, or steam pretreatment or enzymatic hydrolysis or fermentation can increase its nutrient content and suitability as a feed additive for monogastric animals. However, it is only chemical and biological treatments of PKC that seem to improve its nutrient value [4].

In poultry, cecal bacteria can in part digest the NSPs from energy sources such as wheat [5,6,7] and protein sources as PKC [8]. An even better digestibility of PKC is accomplished by addition of relevant exogenous enzymes on low shell PKC [8]. The cell walls or NSP fiber content of PKC consists of 77–79% galactomannan, 12% cellulose and approximately 3% each of arabinoxylan and glucoronoxylan [9]. The galactomannans in PKC consist of a (1→4)-linked β-mannose backbone; with a low degree (12–20%) of α-galactose substituted in a (1→6)- linkage [10]. Endo-β(1→ 4)-mannanases (endomannanases) catalyse solubilisation of β-mannans present in plant cell walls to mannan oligosaccharides (MOS). MOS are prebiotic and implicated in various biological functions, especially in enhancing the immune response, decreasing gut pathogens including *E.coli* [8] and *Salmonella* spp. as well as increasing the diversity of beneficial gut microorganisms in poultry [11,12,13].

Although the commercial use of fiber degrading feed enzymes has been well known since the 1980s [14,15], the global market for feed enzymes is continually increasing, substantially motivated by the growing concern regarding animal health and the need to maximise the nutrient uptake from feed. It is estimated to account for USD 1.3 billion in 2020 and is projected to reach USD 1.9 billion by 2025 [16]. These figures justify efforts to continue researching more efficient enzymes.

Although inclusion levels of PKC in poultry diets have been studied by several researchers [17,18,19], there seems to be a general disagreement on the recommended levels of inclusion. Utilized amounts of PKC vary from one study to another [18], most likely due to lack of proper systematic documentation as well as addition of different kinds of enzymes including NSPase such as mannanase alone or, for example, together with xylanases, proteases and galactosidases [20] in the different studies. One of the ways of circumventing this variation would be to document the efficacy of a relevant enzyme product thoroughly instead of concluding inclusion levels of PKC from several studies using either a single enzyme or cocktail or blends of enzymes. Ideally, an in vivo set up would be the most relevant way to produce reliable data, but such trials are extremely expensive. In addition, ethical constraints on experimentation with animals must be taken into consideration. In this study, we therefore conduct a thorough in vitro study of a commercial mono component β-mannanase on PKC to support the good in vivo effects using β-mannanase and justify the use of higher levels of PKC to decrease the dependency on use of SBM as the only protein source in animal feed rations. The ability of an endo mannanase to solubilize mannan in PKC and to produce mannan oligomers was studied by nuclear magnetic resonance (NMR) and colorimetry (DNS (3,5-Dinitrosalicylic acid) assay). Using confocal immuno-microscopy, the action of the β-mannanase on PKC was also visualized.

## 2. Results

### 2.1. Analysis of PKC Used

The PKC used in this study contained 35.6% crude fiber, 13.9% of crude protein, 3.6% moisture, 3.9% ashes, 6% ether extract and 37% nitrogen-free extract. Ezieshi and Olomu report comparable amounts of ashes, ether extract and crude protein, but lower levels (10–18%) of crude fiber [21] in three types of African PKC. Also Düsterhöft et al. [9], Cervero et al. [22] and Azizi et al. [3] report similar levels of crude protein and ether extracts, while crude fiber content is not reported in a comparable way.

**Figure 1:**
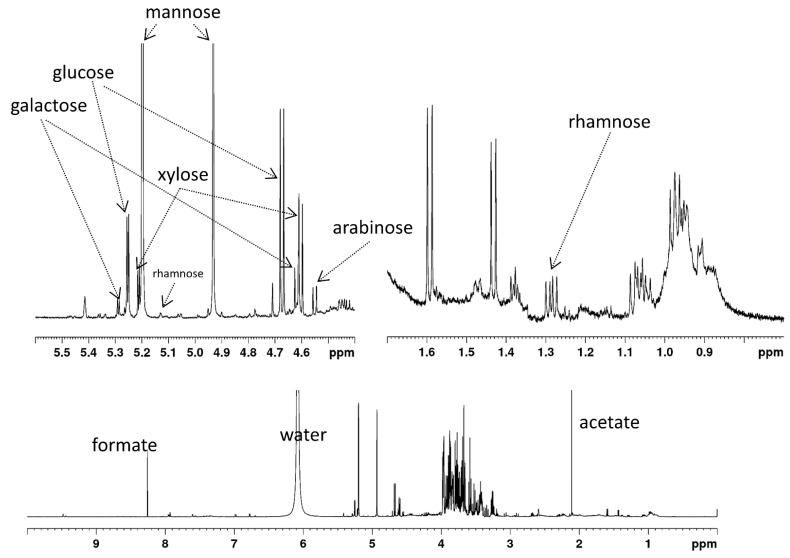
^1^H-NMR spectrum of acid-hydrolyzed PKC, recorded at 280 K in 10% D_2_SO_4_ in D_2_O. The lower panel shows the complete spectrum, the upper left panel shows a zoom of the region displaying the resonances of the anomeric hydrogen atoms, the upper right panel shows a zoom of the methyl group region.

Table 1 gives the content of six monosaccharides in the material. Figure 1 shows the ^1^H-NMR spectrum of hydrolyzed PKC that was used to calculate the monosaccharide composition.

The monosaccharide content is comparable to that found by Cervero et al. [22], who found 17.1% of mannose (*w*/*w* of total PKC) and 3.9% of glucose and did not report any other monosaccharides. Düsterhöft et al. [9] also report small amounts of galactose, xylose, arabinose and rhamnose in PKC.

### 2.2. Enzymatic Digestion

A total reducing sugar assay (DNS assay) was performed on all samples after incubation with the β-mannanase. Enzymatic treatment of PKC leads to a clear increase in reducing sugar content. Table 2 shows the resulting average absorbance readings.

**Figure 2:**
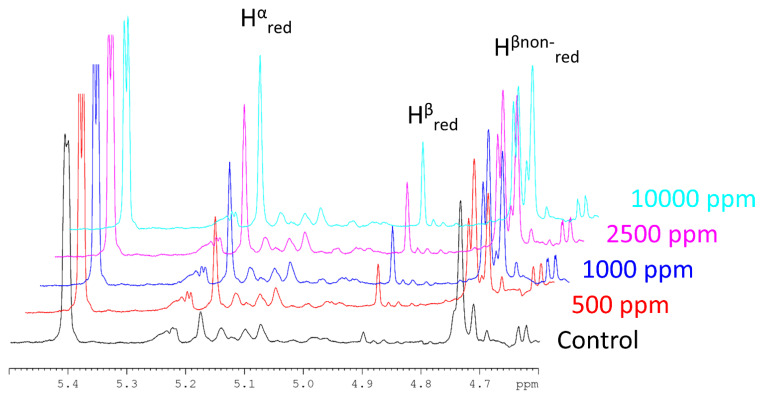
^1^H-NMR spectra of anomeric region of mannan. The control sample is shown in black. Colored spectra show the sample after treatment with different concentrations of *B. subtilis* mannanase. 1 ppm equals 1 µg of commercial enzyme preparation per kg of PKC. The commercial dosage is 500 ppm. The NMR resonances used for integration are marked: H^α^_red_ denotes the anomeric hydrogen atom of the reducing end mannose moiety in α-configuration, H^β^_red_ denotes the anomeric hydrogen atom of the reducing end mannose moiety in β-configuration and H^β^_nonred_ denotes the anomeric hydrogen atoms of all other mannose units.

**Figure 3:**
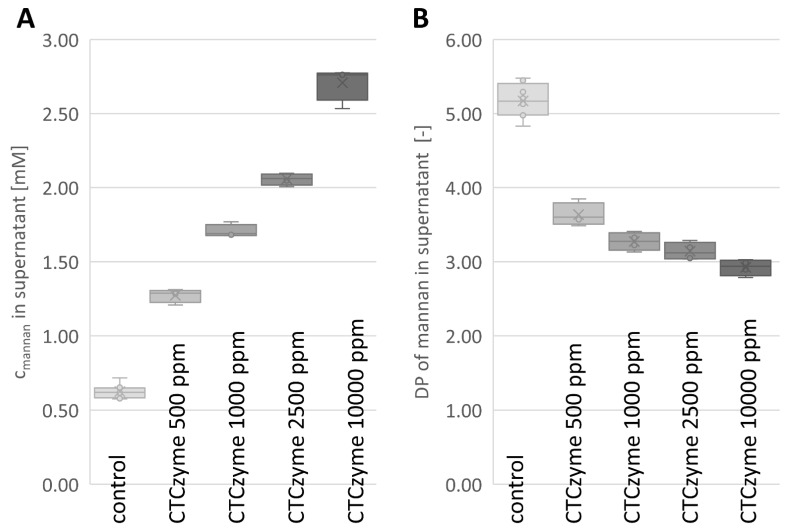
Characterization of mannans released by enzymatic treatment: (**A**): concentration of soluble mannans in treated samples. (**B**): average degree of polymerization (DP) of soluble mannans in control and treated samples.

Examples for a set of ^1^H-NMR spectra of treated PKC are shown in Figure 2, illustrating the increase in MOS peaks because of enzymatic treatment. From ^1^H-NMR, the concentration of MOS (sum of all soluble MOS) and their average DP in the samples was calculated using Equations (1) and (2), andthe results are given in Table 2 and Figure 3.

With the value given in Table 1 for the content of anhydrous mannose in our PKC (11.5 % (*w*/*w*)), we can calculate that 1.2 g of the PKC used contain 0.138 g of mannan. The total mass of mannan solubilized can be calculated using Equations (3)–(5). Equation (7) can be used to determine the fraction of total mannose solubilized by enzymatic treatment. These results are shown in Table 3.

### 2.3. Microscopy

**Figure 4:**
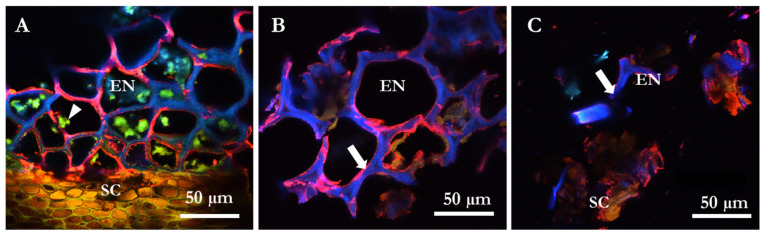
Confocal overlay images of cross sections from palm kernel cake (PKC) labelled with the immunofluorescent probe LM 21 detecting β-mannans (red signal) and calcofluor white dye staining cellulose containing β-glucan linkages (blue signal). Panel (**A**) shows the endosperm cells (EN) with content of protein bodies (arrow head) and the seed coat (SC) in a control section. In panel (**B**) the localization of β-mannan and β-glucan is seen within the intact thick-walled EN (arrow) in a control section. Panel (**C**) shows the disruption of cell wall structures (arrow) after treatment with a commercial dosage of a β–mannanase.

The specific degradation of cell walls structures of PKC by a β-mannanase was studied using monoclonal antibody LM21 and calcofluor dye with confocal microscopy. The micrographs showed that there was an overlap of β-mannan LM 21 epitope and β-glucan structures (hemicellulose and cellulose, respectively) in the thick-walled endosperm cells of PKC (Figure 4). A decrease in the red fluorescence signal is the result of disappearance of the epitopes of the cell walls due to solubilization of β-mannan (Figure 4C) as compared to the control sample (Figure 4B). No unspecific binding of antibodies was observed in the negative control samples labelled only with secondary antibody goat α-rat Alexa-555 (not shown). There was also disappearance of the β-glucan signal as seen by a decrease in the blue color of calcofluor (Figure 4C) indicating disruption of the entire cell wall on using commercially recommended dosages of the β-mannanase.

Immuno-micrographs (Figure 4A) clearly visualize endosperm with numerous protein bodies surrounded by the thick cell wall matrix. The phenol rich brown seed coat layer is also visualized. Figure 4B is representative of a control sample of PKC showing intact cell wall structures (white arrow) while Figure 4C is a representative of β-mannanase treated sample showing a notable dissolution and loss of cell wall structure (white arrow) loosening the endosperm and seed coat as well.

Microscopy data from cell walls clearly shows a clear overlapping of the red signal from mannan and the blue signal from cellulose visualized as a pink signal output (Figure 4A,B). With the solubilization of the mannan by the B. subtilis β-mannanase product, there is a loosening of the compact cell wall structure, and a visible access to the protein within the cell walls can be seen using both commercial (Figure 4C) as well as 2 times commercial dosages (data not shown).

## 3. Discussion

DNS or reducing ends assay measurements are routinely used to determine the activity of carbohydrases or NSPases against various polysaccharides [23,24]. Bååth et al. used reducing ends to obtain an overall picture of the activity of two mannanases on three different mannan substrates [25], spruce O-acetyl-galactoglucomannan, konjac glucomannan and locust bean gum galactomannan. The same measurement was used in our work to evaluate the overall capability of the β-mannanase to solubilise polysaccharides present in the PKC cell walls (Table 2). The produced oligosaccharides were detected and quantified by NMR.

An increase in solubilized mannan was measured with ^1^H NMR upon reaction of the β-mannanase with PKC. As a prebiotic, manno-oligosaccharides (MOS) have been shown to increase proliferation of probiotic bacteria [26,27]. Kalidas et al. have shown that *Lactobacillus reuteri* C1, a probiotic isolated from chicken gut [28], prefers to grow on MOS from PKC with a DP of 3 or 4. Based on this, we speculate that the investigated *B. subtilis* β-mannanase (CTCzyme) would help to proliferate probiotic beneficial bacteria in the poultry gut, as the average DP of MOS produced by the β-mannanase is below 4 at the recommended commercial dosage.

An increasing enzyme concentration leads to an increase of the released soluble mannan concentration (Figure 2, Table 3). However, the increase in soluble mannan concentration does not increase linearly with the enzyme concentration. This may in part be due to a limitation of mannan sites accessible to the enzyme. In addition, some enzyme activity will lead to the further breakdown of already solubilized MOS rather than solubilizing the β-mannan polymer to additional MOS. On incubation of PKC (2%) at pH 5, 50 °C for 12 h with β-mannanase (0.5 U/mL) from *A. oryzae,* Jana and Kango obtained 2.05 mg/mL of MOS [29]. Both M_2_ and M_3_ MOS were produced, of which M_2_ was the major MOS. The substrate concentration used in the same study was 5 times lower and the enzymatic activity of the solution used was 12 times higher than the enzymatic activity used in the current study. In our case, using a 10% PKC suspension, we obtained 0.24 ± 0.04 mg/mL of MOS at the recommended commercial dosage of enzyme (0.04 U/mL). The incubation time in the current study was 4 h at 40 °C, compared to 12 h at 50 °C in the earlier study [29], which used a 2% PKC suspension and an enzyme concentration more than a factor 12 higher, yet reported only 8 times the amount of MOS compared to our study.

The reaction conditions of 4 h incubation with commercial dosage of enzyme at 40 °C used in this study is representative of the time and temperature the enzyme will experience in the GI tract of non-ruminants when used as a feed additive. Thus, our data shows that the *B. subtilis* β-mannanase in the current study is superior to the β-mannanase from *A. oryzae* and most likely can be used to predict the performance of the enzyme in vivo.

Microscopy of PKC shows intact cell walls despite the processing of palm kernel to remove the oil processed (Figure 2 and Figure 4). Mannans in cell walls are NSP heteroglycan fiber components having both storage and structural functions [30,31]. The H-bond network linking mannan polysaccharides to cellulose [32] can only be destroyed under in vitro conditions by use of guanidinium thiocyanate or KOH of high molarity [32]. Cellulose is an important constituent of the NSP fraction in PKC [10,33]. Microscopy data from cell walls clearly shows overlapping of the red signal from mannan and the blue signal from cellulose visualized as a pink signal output. (Figure 4A,B). With the solubilization of the mannan by the *B. subtilis* β-mannanase, there is a loosening of the compact cell wall structure, and a visible access to the protein within the cell walls can be seen using both commercial (Figure 4C) as well as for 2-, 5- and 20-times commercial dosages (data not shown). It is evident as seen with the microscopy pictures that *B. subtilis* β-mannanase at commercial dosage degrades the galactomannan present in PKC cell walls which most likely increases the availability of contained protein to either exo- or endogenous proteases. Most of the microscopy work in the literature has been conducted with 100 to 1000× commercial dosage of NSP degrading enzymes [34,35]. The microscopy experiments performed in this piece of work required only commercial dosages of enzyme indicating the high efficacy of the monocomponent *B. subtilis* β-mannanase enzyme used towards PKC substrate.

Mannan oligosaccharides (MOSs), both those originating from yeast as well as plant derived, despite their structural differences are often referred to as one of the potential alternatives to replace antimicrobial growth promoters in poultry; they canbind the threadlike fimbriae on pathogenic bacteria, preventing them from attaching to the intestinal wall. These MOS are in the category of products called prebiotics [36]. They are non-viable, non-digestible carbohydrate ingredients having a degree of polymerization (DP) of 2–9 and when supplemented in diets in very small amounts allow for specific modulation of both the composition of and/or activity of the gastrointestinal microflora conferring benefits upon hosts’ well-being and health [36,37]. Literature reports have shown that yeast cell wall derived MOS [38,39] and plant β-mannan oligosaccharides [40,41,42] effectively decreased growth of pathogenic bacteria. During an infection process, bacteria colonize the intestinal epithelia mucosa by attachment via their Type-1 fimbriae. MOS can also agglutinate bacteria via their Type-1-fimbriae, resulting in a lower intestinal colonization of these pathogenic bacteria [43]. Zang et al. produced MOS having DP of 2–5 from locust bean gum mannan using a bacterial *Bacillus pumilus* GBSW19 β-mannanase [44]. These MOS significantly enhanced generation of signaling molecules such as intracellular Ca^2+^ and reactive oxygen species (ROS) in plants in a series of reactions eventually leading to prevention of pathogen invasion. MOS obtained from PKC using *Aspergillus oryzae* β-mannanase showed highest cytotoxicity (74.19%) against human colon adenocarcinoma cell line [29]. In another study, Partridge Shank chickens were administered MOS produced from *Amorphophallus konjac* by β-mannanase from *Aspergillus niger* [45]. The study showed improved immune function and intestinal oxidative status as well as reduced cecal *Salmonella* population in the chickens. The authors did not, however, analyze the DP of the MOS generated. Nutrient utilization was improved in a broiler study using the *B. subtilis* β-mannanase used in this study [46]. The same β-mannanase also showed beneficial effects such as decreased cloacal temperature and increased relative thymus weight of broiler chickens raised under hot climatic conditions [47]. Studies by Ryu et al. indicated that dietary supplementation with the same β-mannanase in diets having a high mannan content reversed the adverse effect of the high mannan content on the performance of laying hens [48] and most likely can be used to improve performance and nutrient retention in laying hens. Supplementation of the same β-mannanase enzyme in low energy/low protein diets improved egg production, feed conversion ratio, and apparent ileal digestibility of specific amino acids at peak production of laying hens [49]. Using in vitro methods, we attempt to explain the most likely mechanisms—solubilization of cell walls generating MOS having prebiotic potential—that are responsible for the positive effects of a *B. subtilis* β-mannanase seen in vivo.

## 4. Materials and Methods

### 4.1. Chemicals

All chemicals used were from Sigma-Aldrich (USA), mannan oligosaccharides standards (M_2_ and M_4_) were purchased from Megazyme International, Ireland. Antibodies were purchased from Plant Probes, England and Thermo Fischer, Scientific.

### 4.2. Plant Material

The PKC was obtained from Nutrinor in Colombia as a by-product of Colombian palm oil cultivation.

#### 4.2.1. Determination of Moisture

Moisture was determined as loss on drying following the Association of Official Analytical Chemists (AOAC) official method 930.15 [50] in its revision from March 1999. Briefly, material is weighed, then heated at 135 °C and weighed again.

#### 4.2.2. Determination of Ether Extracts

Ether extract from PKC was determined by the AOAC official method 2003.06 [51]. Briefly, PKC was extracted with hexane, the hexane phase was dried and weighed.

#### 4.2.3. Determination of Crude Protein

Crude protein in PKC was determined following the AOAC official method 990.03 [52]. Briefly, PKC is combusted at 950 °C in pure oxygen atmosphere and nitrogen-containing gases are quantified.

#### 4.2.4. Determination of Crude Fiber

Crude fiber in PKC was determined following the AOAC official method 962.09 [50]. Briefly, PKC is milled, dried, extracted with ether and subsequently boiled for 30 min in 1.25% (*w*/*v*) H_2_SO_4_. The mixture is then filtered and the filter cake is drained for excess water, then boiled for 30 min in 1.25% NaOH. The mixture is then filtered, the filter cake is dried, weighed and then ignited for 30 min at 600 °C, and weighed again. Crude fibre is measured as the loss of weight of the filter cake upon ignition.

Nitrogen-free extract is defined as the remaining content up to 100% after subtraction of moisture, ether extract, crude protein, crude fiber and ashes contents.

### 4.3. Enzyme Product

CTCzyme obtained from CTCBio Inc., South Korea, is a commercial monocomponent β-mannanase product produced by *B. subtilis* with a declaration of 800,000 U/kg mannanase. One enzyme unit is defined as generation of 1 μmole of reducing sugar per min at pH 6.0 and 50 °C using locust bean gum as substrate. CTCzyme is referred to as *B. subtilis* mannanase throughout the article.

### 4.4. Enzyme Treatment of PKC

Samples of PKC were incubated at pH 5 either without enzyme (control) or with the addition of the *B. subtilis* mannanase at commercial (1×), 2×, 5× and 20× the commercial dosage (500 ppm, equivalent to 500 g/ton). In short, 1.2 g of PKC was incubated with 12 mL 0.1 M sodium acetate buffer pH 5 alone (control) or *B. subtilis* β-mannanase product in the same buffer at 40 °C for 4 h with stirring at 500 rpm. After incubation, the samples were centrifuged at 2500× *g* for 10 min. The supernatants were frozen at −80 °C until further analyses. The pellets obtained were washed once with MiliQ water, centrifuged again and dried overnight at 60 °C and used for microscopy.

### 4.5. Colorimetric Assay of Reducing Sugar Content (DNS-Assay)

The DNS reagent was prepared by dissolving 5 g of 3,5-dinitrosalicylic acid in 250 mL of HPLC grade ultra-pure water and adding 100 mL of 2M NaOH to the solution. Potassium sodium tartrate-tetrahydrate (150 g) was then added and the volume of the solution was adjusted to 500 mL with HPLC grade ultra-pure water. The reagent was kept at 4 °C in the dark until used.

Samples were vortexed prior to analysis. 150 μL sample was mixed with 150 μL DNS reagent in a 96-well plate. The plate was covered with Easyseal transparent (Greiner BioOne, Kremsmünster, Austria) and placed in an oven at 105 °C for 15 min. Thereafter, samples were placed on ice immediately to stop the reaction. 50 μL of the reaction mixture were diluted with 250 μL MilliQ-water. Absorbance at 540 nm was measured with a Tecan Spark microplate reader.

A conversion of absorbance to concentrations was not attempted, since it is a well-known fact that the proportionality between absorbance and concentration depends on the saccharide, and the samples contained an unknown composition of saccharides (on top of the mannans potentially released by enzymatic action) [24,53].

### 4.6. Analysis of Mannose Oligosaccharides

All measurements were done on a BRUKER AVIII-600 MHz NMR spectrometer equipped with a 5 mm CPP-TCI probe.

NMR samples were prepared by mixing 500 μL supernatant from enzymatic treatment with 24 μL of D_2_O and 1 μL of a solution of 0.1 M 2,2-Dimethyl-2-silapentane-5-sulfonate sodium salt (DSS), which serves as a chemical shift reference (δ = 0 ppm). Quantitative ^1^H-NMR spectra (20 s recycling delay, 20 ppm spectral width, 65,536 complex datapoints) were recorded at 55 °C to minimize interference of the residual water signal. Absolute quantitation was achieved by the Simple Mixture Analysis Tool of MNova 14.2.0: reference deconvolution was used to determine the integrals of the mannose signals. Integrals were converted to concentration by applying the PULCON method [54] using external 2 mM sucrose as a reference.

The NMR spectra allow for the separate integration of mannose anomeric signals from the reducing end mannose unit (α and β separately) and the anomeric signals from all non-reducing mannose units (all β).

Since all MOS have exactly one reducing end, the total MOS concentration can be calculated by Equation (1), adding the concentrations of α and β reducing-end mannose units, determined from the resonances of H^α^_red_ and H^β^_red_ (see Figure 1).

(1) $$c_{MOS}=c\left(H_{red}^{\alpha }\right)+c\left(H_{red}^{\beta }\right)$$

The average degree of polymerization ($\bar{DP}$), the average number of mannose units per *MOS*) can be calculated by Equation (2), based on the fact that in a *MOS* of *DP* = *n*, there are *n*−1 non-reducing mannose units and one reducing end mannose unit.

(2) $$\bar{DP}=\frac{c\left(H_{nonreducing}^{\beta }\right)}{c_{MOS}}+1$$

The average molecular mass of all soluble *MOS* in the supernatant is given by:

(3) $$\bar{M_{MOS}}=\bar{DP}\left(M_{mannose}-M_{H_{2}O}\right)+M_{H_{2}O}$$

where $M_{mannose}\;$= 180.16 [g/mol] and $M_{H_{2}O}\;$= 18.02 [g/mol]. Thus, the total mass of *MOS* dissolved in the supernatant, $m_{MOS}^{\;}\left[g\right]$, is

(4) $$m_{MOS}^{\;}=\bar{M_{MOS}}*\;c_{MOS}*\;V_{supernatant}$$

where $V_{supernatant}=0.012\;\left[L\right]$ (12 mL).

The total mass of *MOS* solubilized by enzymatic degradation is then obtained by subtracting $m_{MOS}^{\;}$ from the control sample without enzyme added from the $m_{MOS}^{\;}$ from samples with enzyme added.

(5) $$m_{MOS}^{solubilized}=m_{MOS}^{with\;enzyme}-m_{MOS}^{control}\;$$

### 4.7. Immunolocalization and Histology with Confocal Microscopy

Material of PKC was further ground and larger seed coat pieces were removed before fixation in 4% formaldehyde in PBS for 1 h. Samples were washed twice in the buffer and dehydrated in a graded ethanol series (30 min in each step), then gradually infiltrated with melted paraplast (paraffin) using Histochoice clearing agent. The embedding was performed for 2 days at 60 °C. The paraffin blocks were sectioned on a rotary microtome (2030 Biocut microtome Reichert-Jung, AU) to generate 10 μm-thick sections which were adhered to SuperFrost slides. Slides were deparaffinized in pure Histochoice, airdried, and sections were individualized using a PapPen for immunolocalisation studies, performed as in [34]. Sections, from either control samples or samples treated with ß-mannanase enzyme product, were blocked with 5% skimmed milk in PBS for 30 min. Sections were then washed in PBS buffer followed by incubation for 60 min with the rat monoclonal antibody (LM21) diluted 1:10 in the skimmed milk-PBS buffer solution. Samples were subsequently incubated for 1.5 h with the secondary antibody anti-rat IgG linked to an Alexa-555 fluorophore and washed in PBS buffer. A negative control labelling was carried out using only the secondary antibody. Sections were counterstained for 2 min with 0.01% (*w*/*v*) Calcofluor White M2R for identification of ß-glucan linkages such as cellulose, and finally mounted in the anti-fading agent Citiflour AF1 (Agar Scientific, Stanstead, UK). Confocal laser scanning microscopy (CLSM) was done with a TCS SP5x (Leica Microsystems) for visualization of the immuno-label and fluorescent dye. The Alexa555 was excited with the argon laser (488 nm) and the emission range was set to 560–576 nm. Calcofluor White was seen with UV excitation (355 nm) and blue emission (401–443 nm). A 10× objective was used for overview images and a 63× oil immersion objective was used for details. Image cropping, brightness and contrast enhancement were carried out in the LAS AF Lite (Leica) and Adobe Photoshop software.

### 4.8. Total Hydrolysis of PKC, Calculation of Degree of Enzymatic Conversion

PKC was subjected to total hydrolyses by a procedure modified from [55]: at first, 0.1 g of PKC was incubated with 0.1 mL of 72% (*w*/*v*) D_2_SO_4_ in D_2_O at room temperature for 2 h. Subsequently, 620 μL of D_2_O was added to dilute sulfuric acid to 10% (*w*/*v*) and the mixture was placed in a sealed tube and incubated at 121 °C for 2 h. After cooling, the mixture was centrifuged for 10 min at 14,400× *g* and 180 μL of supernatant was removed to a 3 mm-NMR tube to minimize effects of RF-heating [56]. Quantitative ^1^H-NMR spectra were recorded with a recovery delay of 20 s, which is well above 7∙T_1_ of the slowest relaxing nucleus. Molar concentrations of monosaccharides ($c_{monosaccharide}\;\left[\frac{\mathrm{mol}}{L}\right]$) were determined using the Simple Mixture Analysis tool in MNova 14.2.0. This experiment was performed in parallel in quintuplicates.

The total mass of a given monosaccharide in the hydrolyzed PKC was calculated as:

(6) $$m_{monosaccharide}^{anhydrous}=c_{monosaccharide}\;V_{hydrolysis}\left(M_{monosaccharide}-M_{H_{2}O}\right)$$

where $V_{hydrolysis}\;$= 7.2 × 10^−4^ L (720 μL), $M_{monosaccharide}$ is the molar mass of the monosaccharide in question and $M_{H_{2}O}$ is the molar mass of water (18.02 $\left[\frac{g}{\mathrm{mol}}\right]$). The molar mass of water was subtracted to account for the fact that monosaccharides mostly occurred in polymeric chains before hydrolysis. This is indicated by the superscript “*anhydrous*”. Total contents of mannose, glucose, galactose, xylose, rhamnose and arabinose in PKC were thus quantified. The degree of enzymatic conversion could then be obtained by

(7) $$x\left[\%\right]=\frac{m_{MOS}^{solubilized}\;\frac{\bar{M_{MOS}}-M_{H_{2}O}}{\bar{M_{MOS}}}}{m_{mannose}^{anhydrous}}\cdot 100$$

where the mass of solubilized *MOS* is converted to an anhydrous mass and divided by the mass of anhydrous mannan present in PKC.

SAS jmp v16 was used for statistical analysis.

## 5. Conclusions

Using in vitro methods, we have demonstrated that the β-mannanase at commercial dosages can solubilize significant amounts of PKC mannan to MOS. The effect of commercial dosages of the enzyme in solubilizing mannan could also be visualized using microscopy where cell wall dissolution was seen and quantified with nuclear magnetic resonance spectroscopy. The dosages used in this study could be recommended as a guideline for use in vivo. Feeding trials should be performed to determine the optimal dosage, taking into account the additional cost of administering the enzyme.

## Figures and Tables

**Table 1:** The table gives the content of different monosaccharides in % of total PKC and % of total carbohydrate content as determined by total hydrolysis followed by quantitative ^1^H-NMR spectroscopy.

Monosaccharide	$\%\;\left(w/w\right)\;m_{monosaccharide}^{anhydrous}\;\;\mathrm{of}\;\mathrm{Total}\;\mathrm{PKC}{\;}^{a,c}$	% (*w*/*w*) of Total Carbohydrate ^b,c^
mannose	11.53 ± 0.59%	65.2 ± 3.31%
glucose	4.25 ± 0.36%	24.0 ± 2.01%
galactose	0.69 ± 0.10%	3.9 ± 0.54%
xylose	0.84 ± 0.06%	4.8 ± 0.32%
arabinose	0.30 ± 0.02%	1.7 ± 0.11%
rhamnose	0.08 ± 0.03%	0.5 ± 0.18%

^a^ content of different monosaccharides in % of total PKC, calculated by Equation (6). ^b^ the content of different monosaccharides in % of total carbohydrate content. ^c^ results are given as average ± standard deviation of quantifications made in quintuplicates.

**Table 2:** Soluble mannans in enzymatically treated and untreated PKC.

Treatment	Reducing Sugars ^b,c^ A_540_ [a.u.]	C_MOS_^c,i^ in Supernatant [mM]	${\bar{DP}}^{\;c,j}$ [-]
Control	0.37 ± 0.03	0.63 ± 0.04 ^d^	5.17 ± 0.22
*B. subtilis* mannanase 1× ^a^	0.44 ± 0.05	1.27 ± 0.04 ^e^	3.63 ± 0.13
*B. subtilis* mannanase 2× ^a^	0.52 ± 0.02	1.71 ± 0.04 ^f^	3.27 ± 0.10
*B. subtilis* mannanase 5× ^a^	0.60 ± 0.03	2.06 ± 0.03 ^g^	3.14 ± 0.1
*B. subtilis* mannanase 20× ^a^	0.75 ± 0.02	2.71 ± 0.10 ^h^	2.92 ± 0.09

$\bar{DP}$: average degree of polymerization. ^a^ 1×, 2×, 5× and 20× the commercial dosage (500 ppm). ^b^ the measured absorbance at 540 nm observed in the DNS assay applied to the supernatant. ^c^ Results are given as average ± standard deviation of quantifications made in quadruplicates. ^d,e,f,g,h^ these letters indicate that values within the same column are statistically different (Tukey-Kramer *p* < 0.0001). ^i^ the total concentration of solubilized mannan oligosaccharides calculated from quantitative NMR by Equation (1). ^j^ the calculated average degree of polymerization of the mannans present in the sample calculated from quantitative NMR by Equation (2).

**Table 3:** Mannan solubilization by enzymatic treatment.

Treatment	$\bar{M_{MOS}}$ [g/mol] ^b^	$m_{MOS}^{\;}\;[\mathrm{mg}]{\;}^{c,f}$	$m_{MOS}^{solubilized\;}\;[\mathrm{mg}]{\;}^{d}{,}^{f}$	x (% mannan solubilized) ^e,f^
Control	856.28	6.38 ± 0.27	0.0	0.0%
*B. subtilis* mannanase 500 ppm ^a^	606.59	9.27 ± 0.28	2.89 ± 0.43	1.82 ± 0.27%
*B. subtilis* mannanase 1000 ppm ^a^	540.11	11.06 ± 0.24	4.68 ± 0.27	2.95 ± 0.17%
*B. subtilis* mannanase 2500 ppm ^a^	509.30	12.57 ± 0.21	6.20 ± 0.15	3.89 ± 0.09%
*B. subtilis* mannanase 10,000 ppm ^a^	486.60	15.81 ± 0.59	9.44 ± 0.79	5.91 ± 0.50%

^a^ 1 ppm equals 1 µg of commercial enzyme preparation per kg of PKC. The commercial dosage is 500 ppm. ^b^ The average molecular mass of soluble mannan oligosaccharides ($\bar{M_{MOS}}$) as calculated by Equation (3). ^c^ the total mass of MOS present as calculated by Equation (4). ^d^ the total mass of MOS solubilized as calculated by Equation (5). ^e^ the corresponding degree of mannose solubilization using the total mannan content of 0.136 g in the sample used for enzymatic treatment as calculated by Equation (7). ^f^ Given as mean ± standard deviation of four replicates.

## Data Availability

Not applicable.

## References

[1] FAO (2020). Food Outlook—Biannual Report on Global Food Markets.

[2] Heuzé V., Tran G., Sauvant D., Noblet J., Renaudeau D., Bastianelli D., Lebas F. (2016). Palm kernel meal. Feedipedia, a Programme by INRAE, CIRAD, AFZ and FAO.

[3] Azizi M.N., Loh T.C., Foo H.L., Chung E.L.T. (2021). Is palm kernel cake a suitable alternative feed ingredient for poultry?. Animals.

[4] Sharmila A., Alimon A., Azhar K., Noor H. (2014). Improving nutritional values of palm kernel cake (PKC) as poultry feeds: A review. Malays. J. Anim. Sci..

[5] Choct M., Annison G. (1990). Anti-nutritive activity of wheat pentosans in broiler diets. Br. Poult. Sci..

[6] Choct M., Hughes R.J., Bedford M.R. (1999). Effects of a xylanase on individual bird variation, starch digestion throughout the intestine, and ileal and caecal volatile fatty acid production in chickens fed wheat. Br. Poult. Sci..

[7] Józefiak D., Rutkowski A., Martin S. (2004). Carbohydrate fermentation in the avian ceca: A review. Anim. Feed. Sci. Technol..

[8] Navidshad B., Liang J.B., Jahromi M.F., Akhlaghi A., Abdullah N. (2016). Effects of enzymatic treatment and shell content of palm kernel expeller meal on performance, nutrient digestibility, and ileal bacterial population in broiler chickens. J. Appl. Poult. Res..

[9] Düsterhöft E.M., Voragen A.G.J., Engels F.M. (1991). Non-starch polysaccharides from sunflower (*Helianthus annuus*) meal and palm kernel (*Elaeis guineensis*) meal-preparation of cell wall material and extraction of polysaccharide fractions. J. Sci. Food Agric..

[10] Düsterhöft E.M., Posthumus M.A., Voragen A.G.J. (1992). Non-starch polysaccharides from sunflower (*Helianthus annuus*) meal and palm-kernel (*Elaeis guineensis*) meal—Investigation of the structure of major polysaccharides. J. Sci. Food Agric..

[11] Van der Wielen P.W.J.J., Biesterveld S., Notermans S., Hofstra H., Urlings B.A.P., van Knapen F. (2000). Role of Volatile Fatty Acids in Development of the Cecal Microflora in Broiler Chickens during Growth. Appl. Environ. Microbiol..

[12] Shashidhara R.G., Devegowda G. (2003). Effect of dietary mannan oligosaccharide on broiler breeder production traits and immunity. Poult. Sci..

[13] Rezaei S., Faseleh Jahromi M., Liang J.B., Zulkifli I., Farjam A.S., Laudadio V., Tufarelli V. (2015). Effect of oligosaccharides extract from palm kernel expeller on growth performance, gut microbiota and immune response in broiler chickens. Poult. Sci..

[14] Hesselman K., Åman P. (1986). The effect of β-glucanase on the utilization of starch and nitrogen by broiler chickens fed on barley of low- or high-viscosity. Anim. Feed. Sci. Technol..

[15] Pettersson D., Aman P. (1989). Enzyme supplementation of a poultry diet containing rye and wheat. Br. J. Nutr..

[16] Rizvi S. (2020). Feed Enzymes Market by Type (Phytase, Carbohydrase, and Protease), Livestock (Poultry, Swine, Ruminants, and Aquatic Animals), Source (Microorganism, Plant, and Animal), Form (Dry and Liquid), and Region—Global Forecast to 2025. https://www.researchandmarkets.com/reports/5129255/feed-enzymes-market-by-type-phytase?utm_source=GNOM&utm_medium=PressRelease&utm_code=qj4fhb&utm_campaign=1428743+-+Global+Feed+Enzymes+Industry+Report+2020-2025%3a+Dominated+by+BASF%2c+DowDuPont%2c+Koninklijke+DSM%2c+Kemin+Industries%2c+and+Cargill+Incorporated&utm_exec=joca220prd.

[17] Onwudike O.C. (1986). Palm kernel meal as a feed for poultry. 2. Diets containing palm kernel meal for starter and grower pullets. Anim. Feed Sci. Technol..

[18] Chong C.H., Zulkifli I., Blair R. (2008). Effects of dietary inclusion of palm kernel cake and palm oil, and enzyme supplementation on performance of laying hens. Asian Australas. J. Anim. Sci..

[19] Matos N., Polanco R., De Jesus C., Vasquez R. Effects of palm kernel cake on daily gain and carcass yield of broiler chicks. Proceedings of the Caribbean Food Crops Society > 44th Annual Meeting.

[20] Soltan M.A. (2009). Growth performance, immune response and carcass traits of broiler chicks fed on graded levels of palm kernel cake without or with enzyme supplementation. Livest. Res. Rural Dev..

[21] Emeka V.E., Julius M.O. (2007). Nutritional evaluation of palm kernel meal types: 1. Proximate composition and metabolizable energy values. Afr. J. Biotechnol..

[22] Cerveró J.M., Skovgaard P.A., Felby C., Sørensen H.R., Jørgensen H. (2010). Enzymatic hydrolysis and fermentation of palm kernel press cake for production of bioethanol. Enzym. Microb. Technol..

[23] Breuil C., Saddler J.N. (1985). Comparison of the 3,5-dinitrosalicylic acid and Nelson-Somogyi methods of assaying for reducing sugars and determining cellulase activity. Enzym. Microb. Technol..

[24] Miller G.L. (1959). Use of Dinitrosalicylic acid reagent for determination of reducing sugar. Anal. Chem..

[25] Arnling Bååth J., Martínez-Abad A., Berglund J., Larsbrink J., Vilaplana F., Olsson L. (2018). Mannanase hydrolysis of spruce galactoglucomannan focusing on the influence of acetylation on enzymatic mannan degradation. Biotechnol. Biofuels.

[26] Bello B., Mustafa S., Tan J.S., Ibrahim T.A.T., Tam Y.J., Ariff A.B., Manap M.Y., Abbasiliasi S. (2018). Evaluation of the effect of soluble polysaccharides of palm kernel cake as a potential prebiotic on the growth of probiotics. 3 Biotech.

[27] Li Y.X., Liu H.J., Shi Y.Q., Yan Q.J., You X., Jiang Z.Q. (2020). Preparation, characterization, and prebiotic activity of manno-oligosaccharides produced from cassia gum by a glycoside hydrolase family 134 β-mannanase. Food Chem..

[28] Kalidas N.R., Saminathan M., Ismail I.S., Abas F., Maity P., Islam S.S., Manshoor N., Shaari K. (2017). Structural characterization and evaluation of prebiotic activity of oil palm kernel cake mannanoligosaccharides. Food Chem..

[29] Jana U.K., Kango N. (2020). Characteristics and bioactive properties of mannooligosaccharides derived from agro-waste mannans. Int. J. Biol. Macromol..

[30] Liepman A.H., Nairn C.J., Willats W.G.T., Sørensen I., Roberts A.W., Keegstra K. (2007). Functional genomic analysis supports conservation of function among cellulose synthase-like a gene family members and suggests diverse roles of mannans in plants. Plant Physiol..

[31] Scheller H.V., Ulvskov P. (2010). Hemicelluloses. Annu. Rev. Plant Biol..

[32] Schröder R., Atkinson R.G., Redgwell R.J. (2009). Re-interpreting the role of endo-β-mannanases as mannan endotransglycosylase/hydrolases in the plant cell wall. Ann. Bot..

[33] Daud M.J., Jarvis M.C. (1992). Mannan of oil palm kernel. Phytochemistry.

[34] Pedersen N.R., Ravn J.L., Pettersson D. (2017). A multienzyme NSP product solubilises and degrades NSP structures in canola and mediates protein solubilisation and degradation in vitro. Anim. Feed Sci. Technol..

[35] Ravn J.L., Martens H.J., Pettersson D., Pedersen N.R. (2015). Enzymatic solubilisation and degradation of soybean fibre demonstrated by viscosity, fibre analysis and microscopy. J. Agric. Sci..

[36] Gibson G.R., Roberfroid M.B. (1995). Dietary modulation of the human colonic microbiota: Introducing the concept of prebiotics. J. Nutr..

[37] Pineiro M., Asp N.G., Reid G., Macfarlane S., Morelli L., Brunser O., Tuohy K. (2008). FAO Technical meeting on prebiotics. J. Clin. Gastroenterol..

[38] Oyofo B.A., DeLoach J.R., Corrier D.E., Norman J.O., Ziprin R.L., Mollenhauer H.H. (1989). Prevention of Salmonella typhimurium colonization of broilers with D-mannose. Poult. Sci..

[39] Spring P., Wenk C., Dawson K.A., Newman K.E. (2000). The effects of dietary mannanoligosaccharides on cecal parameters and the concentrations of enteric bacteria in the ceca of salmonella-challenged broiler chicks. Poult. Sci..

[40] Agunos A., Ibuki M., Yokomizo F., Mine Y. (2007). Effect of dietary **β** 1–4 mannobiose in the prevention of *Salmonella enteritidis* infection in broilers. Br. Poult. Sci..

[41] Ishihara N., Chu D.C., Akachi S., Juneja L.R. (2000). Preventive effect of partially hydrolyzed guar gum on infection of Salmonella enteritidis in young and laying hens. Poult. Sci..

[42] Jahromi M.F., Shokryazdan P., Idrus Z., Ebrahimi R., Bashokouh F., Liang J.B. (2017). Modulation of immune function in rats using oligosaccharides extracted from palm kernel cake. Biomed. Res. Int..

[43] Ofek I., Mirelman D., Sharon N. (1977). Adherence of *Escherichia coli* to human mucosal cells mediated by mannose receptors. Nature.

[44] Zang H., Xie S., Zhu B., Yang X., Gu C., Hu B., Gao T., Chen Y., Gao X. (2019). Mannan oligosaccharides trigger multiple defence responses in rice and tobacco as a novel danger-associated molecular pattern. Mol. Plant Pathol..

[45] Zhou M., Tao Y., Lai C., Huang C., Zhou Y., Yong Q. (2019). Effects of mannanoligosaccharide supplementation on the growth performance, immunity, and oxidative status of partridge shank chickens. Animals.

[46] Mussini F.J., Coto C.A., Goodgame S.D., Lu C., Karimi A.J., Lee J.H., Waldroup P.W. (2011). Effect of ß-mannanase on broiler performance and dry matter output using corn soybean meal based diets. Int. J. Poult. Sci..

[47] Yang T.S., Kim M.C., Martinez-Pitargue F., Choi H.S., Kil D.Y. (2019). Dietary β-mannanase decreases cloacal temperature of broiler chickens under hot conditions without affecting growth performance. Rev. Colomb. Ciencias Pecu..

[48] Ryu M.H., Hosseindoust A., Kim J.S., Choi Y.H., Lee S.H., Kim M.J., Lee J.H., Chae B.J. (2017). β-mannanase derived from Bacillus Subtilis WL-7 improves the performance of commercial laying hens fed low or high mannan-based diets. J. Poult. Sci..

[49] White D., Adhikari R., Wang J., Chen C., Lee J.H., Kim W.K. (2021). Effects of dietary protein, energy and β-mannanase on laying performance, egg quality, and ileal amino acid digestibility in laying hens. Poult. Sci..

[50] AOAC Official Methods. http://www.aoacofficialmethod.org.

[51] Thiex N.J., Anderson S., Gildemeister B., Adcock W., Boedigheimer J., Bogren E., Coffin R., Conway K., DeBaker A., Frankenius E. (2003). Crude fat, hexanes extraction, in feed, cereal grain, and forage (Randall/Soxtec/Submersion method): Collaborative Study. J. AOAC Int..

[52] Sweeney R.A. (1989). Generic combustion method for determination of crude protein in feeds: Collaborative study. J. AOAC Int..

[53] McCleary B.V., McGeough P. (2015). A Comparison of polysaccharide substrates and reducing sugar methods for the measurement of endo-1,4-β-Xylanase. Appl. Biochem. Biotechnol..

[54] Wider G., Dreier L. (2006). Measuring protein concentrations by NMR spectroscopy. J. Am. Chem. Soc..

[55] De Souza A.C., Rietkerk T., Selin C.G.M., Lankhorst P.P. (2013). A robust and universal NMR method for the compositional analysis of polysaccharides. Carbohydr. Polym..

[56] Wimmer R., Wider G. (2007). Real-time imaging of the spatial distribution of rf-heating in NMR samples during broadband decoupling. J. Magn. Reson..

